# Autonomous Rear Parking via Rapidly Exploring Random-Tree-Based Reinforcement Learning

**DOI:** 10.3390/s22176655

**Published:** 2022-09-02

**Authors:** Saugat Shahi, Heoncheol Lee

**Affiliations:** Department of IT Convergence Engineering, Kumoh National Institute of Technology, Gumi 39177, Korea

**Keywords:** autonomous rear parking, OpenAI Gym, path planning, path following, model predictive control, reinforcement learning

## Abstract

This study addresses the problem of autonomous rear parking (ARP) for car-like nonholonomic vehicles. ARP includes path planning to generate an efficient collision-free path from the start point to the target parking slot and path following to produce control inputs to stably follow the generated path. This paper proposes an efficient ARP method that consists of the following five components: (1) OpenAI Gym environment for training the reinforcement learning agent, (2) path planning based on rapidly exploring random trees, (3) path following based on model predictive control, (4) reinforcement learning based on the Markov decision process, and (5) travel length estimation between the start and the goal points. The evaluation results in OpenAI Gym show that the proposed ARP method can successfully be used by minimizing the difference between the reference points and trajectories produced by the proposed method.

## 1. Introduction

The tremendous increase in the number of vehicles has influenced traffic and mobility. Autonomous vehicles (AVs) have proven to be a solution for overcoming traffic problems [1]. In terms of the path planning and movement of AVs, significant achievements have been made. However, autonomous parking remains a concern in terms of smooth path planning, parking slot management, control gain, and motion planning. At the same time, the reverse movement of an AV is a relatively challenging task because of the changes in the control mode. Therefore, an efficient autonomous rear parking (ARP) method is required to fully implement self-controlled AV systems. Several methods have been developed to implement ARP or similar fields in AVs, which are briefly introduced in the next section. This study focuses on the application of reinforcement learning (RL), which has been recently highlighted, to ARP systems.

RL techniques are widely used for intelligent systems because of their learning paradigm and potential to train the agent to act or adapt to the environment. Owing to the extensive development of sensing and computing technologies over time, research in AVs has significantly progressed [2]. In the mid-1990s, INRIA created one of the world’s first experimental prototypes of an automatically parallel-parking electric car called Ligier [3]. In the early 2000s, smart parking sensor technology started to gain popularity, particularly in malls and retail centers [4]. Based on recent statistics, the United States has almost 6000 parking spaces and 25 major automated parking systems (APSs). Simultaneously, Japan is sustaining an estimated 1.6 million APSs [5].

**Figure 1:**
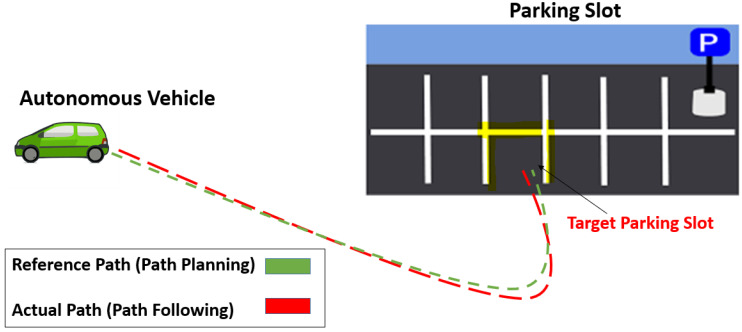
Concept of autonomous rear parking (ARP). The area surrounded by the yellow lines represents the target parking slot. The dotted green and red lines represent the reference path by path planning and the actual path by path following, respectively.

The concept of the ARP system used in this study is illustrated in Figure 1. This involves two steps: path planning to generate a reference path and path following to move along the reference path. The AV acts as an agent for RL, and our path-planning method based on rapidly exploring random trees (RRTs) is employed to generate a reference path used as an expert demonstration to the agent for training purposes. The AV achieves its path-following ability by using our path-following method based on model predictive control (MPC), which determines the motion, position, and speed of the vehicle. RL is used to evaluate the path-planning and path-following abilities of the AV based on the optimal value- and optimal policy-based learning method, which generates a set of rewards and a set of actions for the learning behavior of the reinforcement agent.

The major contributions of this study are summarized as follows: (1) We created a custom OpenAI Gym environment to train the RL agent. (2) We designed an RRT-based path-planning method to create a waypoint for the target parking slot. The primary application of this method is to generate an optimal reference path for the RL agent. (3) We applied MPC to enable an AV to achieve path-following and to calculate the distance, acceleration, and position of the vehicle. (4) We created a Markov decision process (MDP)-based RL method to calculate the value and action required to evaluate the rear parking ability of an AV. Value- and policy-based RL was applied to generate an optimal reward and an optimal action for the agent.

The remainder of this paper is organized as follows. In Section 2, we introduce related studies with a brief comparison and describe the problem of ARP. Section 3 describes the proposed ARP method, which consists of RRT-based path planning, MPC-based path following, MDP-based RL, and distance estimation. Section 4 presents the simulation and evaluation results. Finally, Section 5 presents the conclusions of the study.

## 2. Problem Description

### 2.1. Related Studies

In this section, we highlight some related studies on the ARP of AVs. This review focuses on current research on autonomous parking and the methods implemented to solve this problem, summarized in Table 1. Deep-RL-based trajectory planners for APSs [6] have been developed using neural network architectures and considering the efficiency of human demonstration. However, the efficient and highly precise training of the agent remains an issue. MPC for path following in AVs focuses on control maneuver approaches in dynamic systems [7]. However, vehicle stability and the tracing of the desired trajectory remain challenging. Additionally, a robust controller is required to maintain the speed and position of the AV. The path-planning approach for automatic parking [8] highlights the local and global path-planner framework via multiple methods; however, it is impossible to discuss the motion controller efficiency of the path created by the approach. The MDP framework was introduced to learn proposed driving strategies [9] that evaluate the automatic adaptation to the environment and learning the optimal strategy; however, to determine the strategies, a stochastic model of values or rewards should be defined. In addition, it is quite difficult to examine the learning process without expert demonstration and comparison with reference data.

To resolve the problem of APSs, actor–critic-based RL [16] was introduced with Q-learning with a deep neural network. It achieved better parking slot detection but failed to resolve the path-tracking errors. Path-following control algorithms based on MPC [11] use control techniques for AVs with rear-wheel steering; however, the overall path following is based only on steering control, which requires the precise use of longitudinal and lateral actuators. Deep inverse RL (IRL) [14] was used for the advance planning of an AV using MaxEnt deep IRL. Although the desired driving behavior could be achieved, defining the optimal strategy and policies for AVs is challenging. These expert-like driving behaviors are difficult to achieve owing to the higher computational efficiency required.

A policy-based accelerated deep RL [17] algorithm was proposed for the analysis of policy iterations to optimize the learning rate and accelerate the learning adaptability in both discrete as well as continuous actions. In addition to its practical application in real scenarios, the proposed algorithm is not robust. A model- and neural-controller-based approach [15] was used to highlight the control actions based on sensors and dynamic neural-based processes that optimize the ad hoc performance functions. The resulting model was used to generate the control actions; however, the stability of the AV and robust APS system delivered a poor approach. The sensitivity-based path-following algorithm [18], an approach developed based on MPC, was aimed at improving scenario decomposition for multiple stages. A large-scale optimization problem could be decomposed; however, the uncertain parameters led to errors in non-linear programming.

### 2.2. Autonomous Rear Parking Problem

In the context of AVs, ARP is a concern because AVs are influenced by certain factors, such as the environment, vehicle dynamics, controls, sensors, and decision making. In this case, the forward movement of an AV is dependable and easy to achieve. However, the backward (rear) movement of an AV is challenging. This study focused on resolving the problem of rear parking of AVs. The actual problem with ARP is locating the target parking slot (destination) and defining a path or trajectory, as the path should be smooth and collision-free. Similarly, path navigation is also worth addressing. An OpenAI Gym custom environment was created to depict the ARP problem. The scenario was customized based on the vehicle model, dimensions of the original position of the vehicle, and target parking slot. In addition, a control action was defined to generate a robust controller.

**Figure 2:**
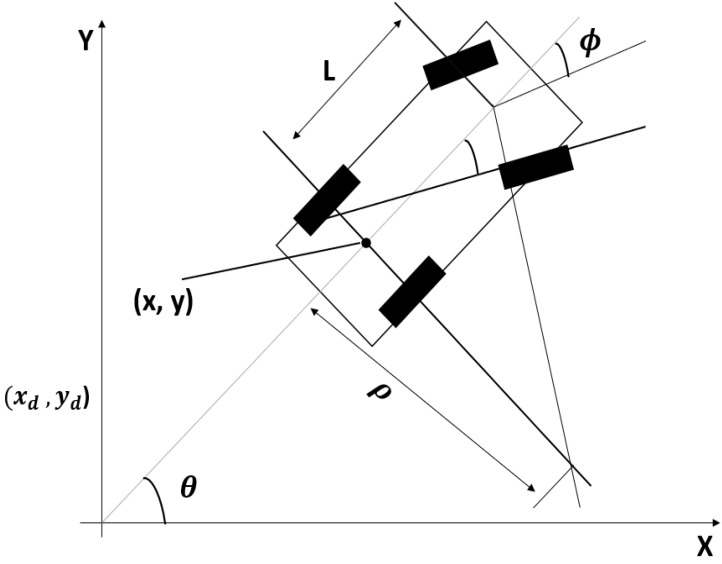
Schematic of the kinematics of car-like four-wheeled vehicles along with their parameters.

The kinematics of a four-wheeled vehicle was provided by de Lope and Maravall [19], as shown in Figure 2, illustrating the concept of the vehicle model, where (*x*, *y*) are the center points of the rear axle; ρ is the radius of the circle; L is the distance between the front and rear axles; and ϕ and θ are the steering angle and direction of vehicle heading, respectively.

Several parameters linked with the car-like four-wheeled vehicle are shown in Figure 2. q = (*x*, *y*, θ) denotes a configuration. The origin of the car is in the middle of the rear axle, and the x-axis runs parallel to the primary axis of the car. The steering angle is denoted as  ϕ; L denotes the distance between the front and rear axles. The car moves in a circular motion if the steering angle is set to ϕ and the radius of the circle is ρ. Factors such as vehicle dynamics, steering angle, and angle of the vehicle with its position must be precise for the control action to generate a motion controller.

## 3. Proposed Method

**Figure 3:**
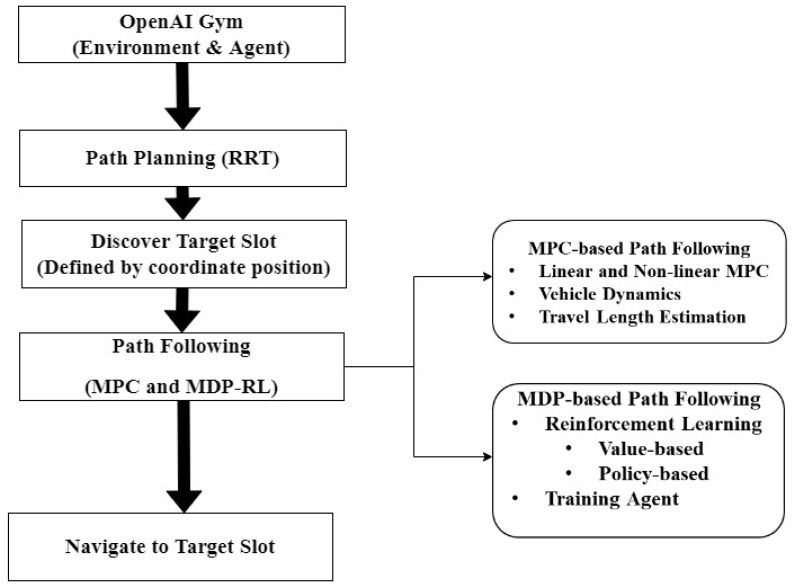
Flowchart of the proposed method, describing the workflow for path planning, path following, and travel length estimation.

Figure 3 shows the workflow of the proposed method for ARP. It also shows the process conducted using the proposed method as the AV routes toward the target parking slot. In the proposed method for ARP, MPC-based path following is used as an expert demonstration for the RL agent (AV). Based on the reference path created by the RRT and expert demonstrations, the agent is trained in an OpenAI Gym environment based on optimal value- and policy-based learning.

### 3.1. OpenAI Gym

OpenAI Gym is an interface used to implement reinforcement learning benchmarks [20]. It provides a suitable environment to develop and test learning agents and offers a wide range to create a customized environment according to our requirements. In the proposed method, OpenAI Gym was used as a toolkit to create a custom ARP environment. The environment was defined using a matrix of [100 × 100], and we created a grid graph to define the coordinates of the starting position of the AVs and the target parking slot. The created environment assisted in efficiently implementing the proposed method by enabling the training of the agent with respect to the state and policies derived from RL.

### 3.2. RRT-Based Path Planning

**Figure 4:**
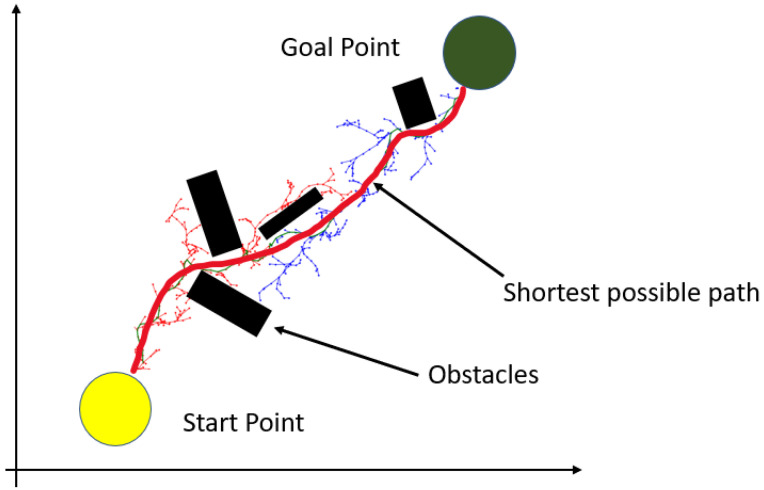
RRT-based path planning to create an optimal reference path. The shortest and best collision-free path is created.

The RRT is an efficient method used to meet the requirements of automatic rear-parking path planning. This is a sampling-based method that creates a search tree from the start point to the destination. In this study, we implemented an RRT to determine the shortest collision-free path from the AV to the target parking slot. First, RRT aims to determine the goal point (target slot) and, second, to draw waypoints. The positions of the vehicle and the parking slot are defined by the array matrix. RRT-based path planning [10] improves the efficiency of identifying the goal point and the path for ARP. As shown in Figure 4, the path created by the RRT is used as a reference path by the RL agent (AV) to train the agent for path following.

### 3.3. MPC-Based Path Following

In this section, we focus on the path following of an AV. This is an online control method used to predict and optimize control actions. This technique defines the path-following controller for an AV [12]. The MPC controller controls vehicle speed and steering, and the AV is directed through the path. As the reference path is generated, the MPC tracks down the path, and the AV navigates to the assigned target parking slot. The position, acceleration, steering, and other factors of an AV are controlled using an MPC path-following controller. MPC is used to improve path following, control gain, vehicle steering, and position of the AV [21]. Two main classes of MPCs exist as shown in Table 2.

**Figure 5:**
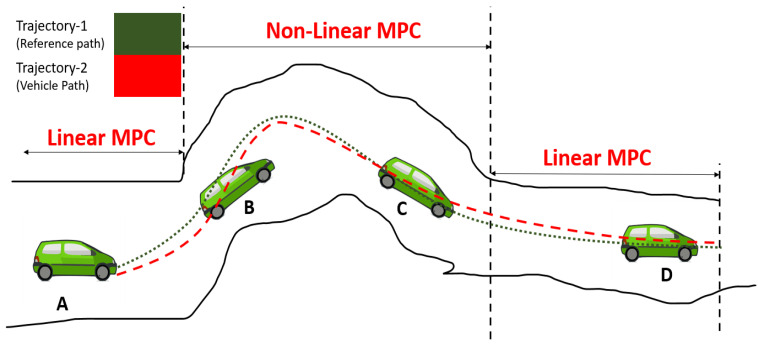
Combined linear and non-linear MPCs. Both techniques are applied in the proposed method.

The concepts of linear and non-linear MPC in the proposed method are shown in Figure 5. Quadratic and nonquadratic functions are defined to generate a stable controller for motion control and path following, respectively.

### 3.4. MDP-Based Reinforcement Learning

The Markov decision process is a mathematical framework in RL. The agent interacts with the ARP environment and collects information about the agent’s state, which generates a value-based reward and a policy-based action [13]. The primary application of MDP is the evaluation of path following via RL. As the agent drives through the path to adapt to the environment, a certain condition or reward is defined. To make a correct decision during path following, a set of policies is defined. In the proposed method, two MDP methods, value- and policy-based learning, were used.

To evaluate the MDP, we implemented Bellman’s equation based on the value and policy iterations. Value, policy, and combined iterations are computed as follows:

(1) $$v_{i+1}^{*}\left(s\right)\leftarrow \max\;a\sum^{}T\left(s,a,s^{\prime }\right)[R\left(s,a,s^{\prime }\right)+\gamma v_{i}^{*}\left(s^{\prime }\right)]$$

(2) $$v_{i+1}^{*}\left(s\right)\leftarrow s^{\prime }\sum^{}T\left(s,\pi \left(s\right),s^{\prime }\right)[R\left(s,\pi \left(s\right),s^{\prime }\right)+\gamma v_{i}^{\pi }\left(s^{\prime }\right)]$$

(3) $$v^{\pi }\left(s\right)\leftarrow s^{\prime }\sum^{}T\left(s,\pi \left(s\right),s^{\prime }\right)[R\left(s,\pi \left(s\right),s^{\prime }\right)+\gamma v_{i}^{\pi }\left(s^{\prime }\right)]$$

Using Equation (3), we can iterate or train the agent with optimal rewards and policies, which is the main application of the MDP in the proposed method.

### 3.5. Estimation of Travel Length

Although path planning and path following are completed, the calculation of the distance or accumulation of waypoints by AVs remains a concern. The interpolation method is an effective approach to overcome this issue. Interpolation typically means the optimal or the best approach. Because the reference and actual paths of the AV slightly diverge, a linear interpolation method is introduced to estimate the travel length from the start point to the target parking slot [22]. In addition, when AVs navigate toward the target parking slot, there are multiple curves; thus, the interpolation method is primarily applied for path smoothing.

The total travel length of the AV is calculated using the Euclidean distance as follows:

(4) $$d=\sqrt{{\left(x_{2}-x_{1}\right)}^{2}+{\left(y_{2}-y_{1}\right)}^{2}}$$

where ($x_{1}$*,* $y_{1}$) and ($x_{2}$*,* $y_{2}$) are the coordinates of the first and second points, respectively.

**Figure 6:**
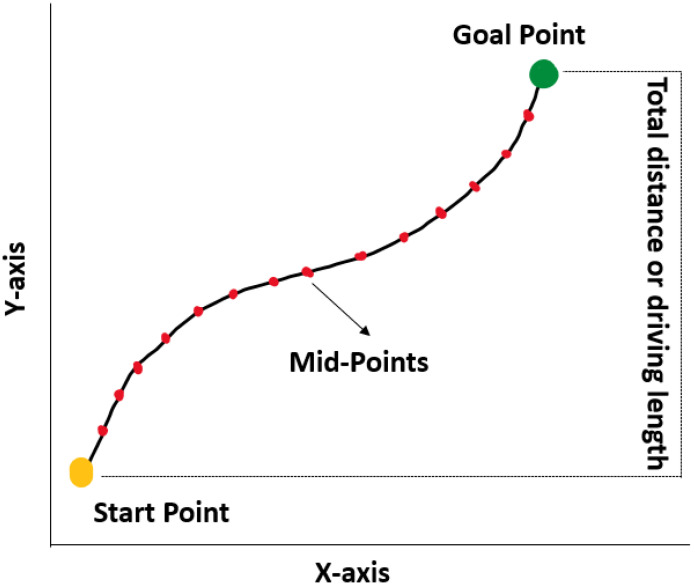
Travel length estimation. Midpoints are used as the checkpoints for the navigation of AVs.

After calculating the distance, the midpoint between each point is calculated to enable navigation of the AV through the desired points that match the reference path. As shown in Figure 6, each midpoint is used by the AV to traverse the path; the midpoints behave as an initial goal point and a start point for the next step. Calculation of the midpoint eases the movement of the AV from one position to another.

(5) $$m=\left[\frac{x_{1}+x_{2}}{2},\frac{y_{1}+y_{2}}{2}\right]$$

## 4. Evaluation Results

**Figure 7:**
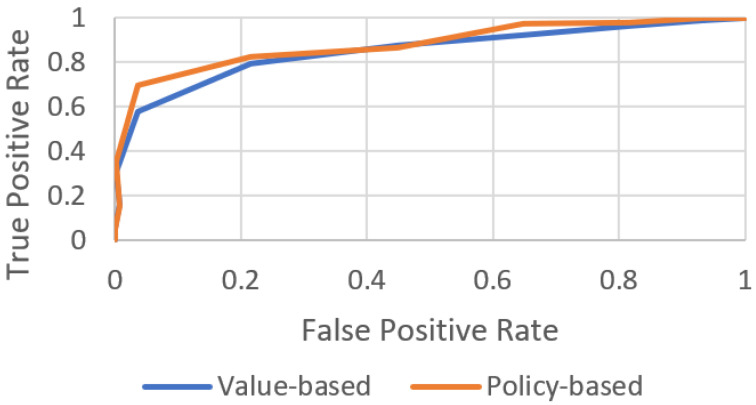
Training process of an agent via RL. The training success rate of the agent is validated based on value and policy.

This section describes the experimental process conducted using the proposed method. It includes the training of agents based on RL, as shown in Figure 7. Value- and policy-based learning, simulation-based experimental results, and the real vehicle test setup are discussed.

**Figure 8:**
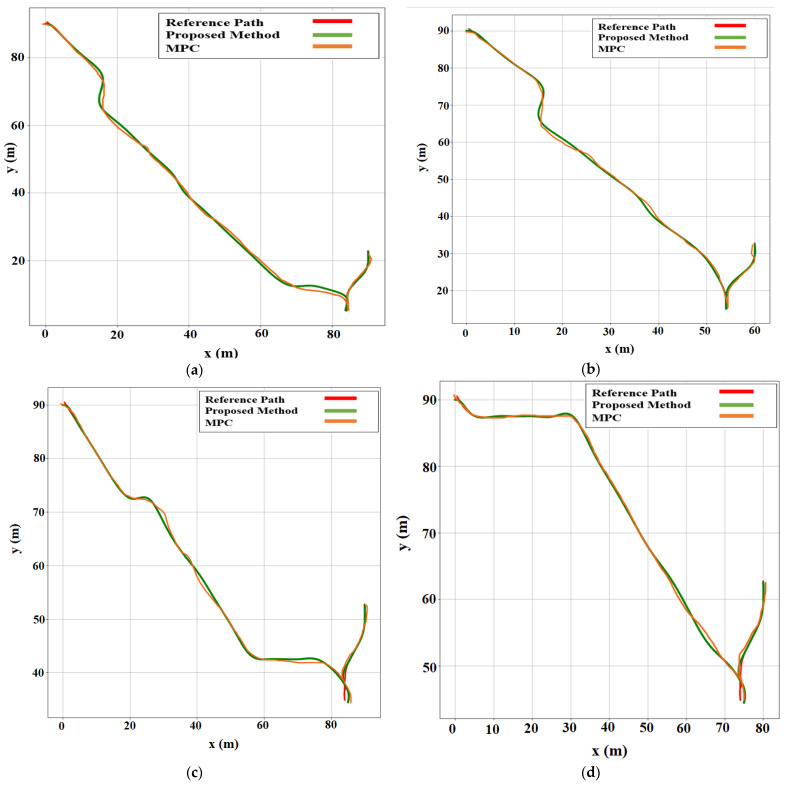

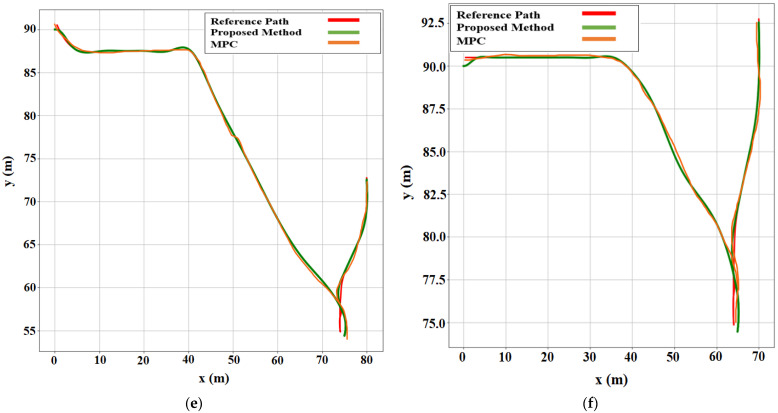
Comparison of path-following techniques with respect to the reference path. For each case, the MPC method was compared with the proposed method. (**a**) Case 1. (**b**) Case 2. (**c**) Case 3. (**d**) Case 4. (**e**) Case 5. (**f**) Case 6.

The simulations and experimental settings were carried out in the OpenAI gym framework using python 3.7.0, where RL acted as a backbone for training the agent. The basic parameters for experimental vehicle and the environment are shown in Table 3. The environment was represented in the form of a graph using coordinate geometry, where (*x*, *y*) = (100, 100). The starting position of AV, the parking and target parking slot, and the path were represented using the coordinate of the graph. The starting position of the AV was constant in all scenarios, i.e., (*x*, *y*) = (0, 90), while the position of the target parking slot was manually input to compare the proposed method in different scenarios and conditions. The starting angle of the AV was parallel to the x-axis, i.e., 180 degrees. We experimented in six different cases, as shown in Figure 8 i.e., Case 1, Case 2, Case 3, Case 4, Case 5, and Case 6 respectively.

### 4.1. Value- and Policy-Based Training

RL was applied to train the agent to adapt to the environment. A typical environmental scenario is shown in Figure 1. RRT-based path-planning methods generate a reference path that acts as an expert path or waypoint for the agent. RL was introduced to evaluate the path-following ability based on MPC.

The value- and policy-based learning techniques based on RL are shown in Figure 7. This indicates that the training process achieved better results. MDP-based RL aims to optimize the learning ability to adapt the reference or expert path by generating the optimal reward function for each state, i.e., the state–reward pair. The chart in Figure 7 shows the reward (value-based) in each state of the reference path as the agent navigates toward the target parking slot.

Policy-based RL determines the optimal policy for each state, i.e., the state–action pair, as shown in Figure 7. This method optimizes the learning behavior of the agent by producing a set of actions (policy-based) in terms of L, R, U, D, and X (left, right, up, down, and exit, respectively).

### 4.2. ARP Results

**Figure 9:**
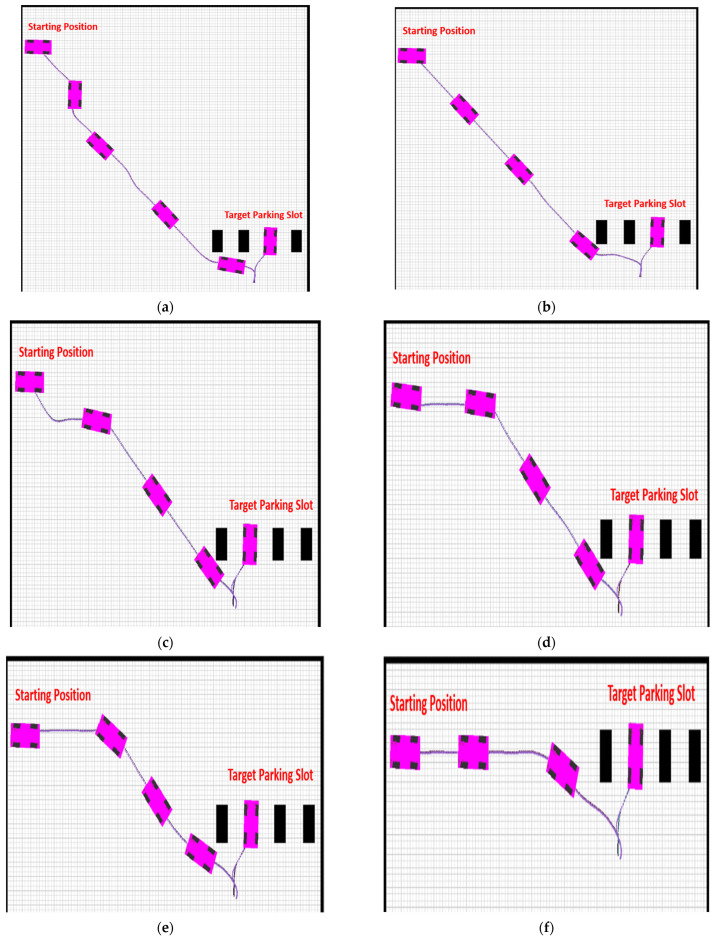
Simulation results in six different scenarios illustrating the proposed ARP system. (**a**) Case 1. (**b**) Case 2. (**c**) Case 3. (**d**) Case 4. (**e**) Case 5. (**f**) Case 6.

The environment setup was established in the OpenAI Gym based on the array matrix, where the position of the AV parking slot and target parking slot were defined as a matrix. The simulation results provided a better approach for automatic rear parking than the other approaches discussed in Section 2.1, as shown in six different environment settings in Figure 9. The errors in the proposed method for the reference path were smaller than those in the MPC-based method.

The quantitative analysis was conducted as follows: According to the path following based on MPC and the proposed method, we calculated the perfection and errors by comparing the reference and actual paths of AVs. Cases 1, 2, 3, 4, 5, and 6 were environments with different parameters. The position of the target parking slot was changed in each case. However, the starting position remained the same for all cases. A comparison between the MPC and the proposed method showed that the proposed method has higher accuracy.

Table 4 shows the quantitative analysis we used to calculate the accuracy using only the MPC method. We used a distance formula based on coordinates to calculate the error distance estimation and calculate the accuracy and error percentage. Table 5 is the analysis of the proposed method used to calculate the accuracy of ARP. We analyzed the calculation in all six cases with different parameters to compare the accuracy of the proposed method versus the MPC-based method. The average accuracy of the proposed method was 94.50%, as shown in Table 5, which was higher than that of the MPC-based method. Moreover, the average error percentage was reduced almost by half to 5.50%. Table 4 and Table 5 show the overall calculation and comparison between the MPC-based method and the proposed method across six different environment settings with different parameters.

The comparison, as shown in Table 6, demonstrates that the proposed method is efficient and has great potential to solve the ARP problem compared with the existing traditional approaches.

Figure 9 shows the actual simulation results for the six different scenarios, which are the cases depicted in Figure 8. The locations and orientations of the vehicle are represented by a graphical car model. As shown in the figure, all the results with the proposed ARP system were successful.

## 5. Conclusions

In this study, we developed a combinational approach based on RL to solve the problem of ARP. The proposed method includes RRT-based path planning, MPC-based path following, MDP, and RL. The overall ARP results and quantitative analysis demonstrated that the proposed method was successful. In addition, the comparison results showed that the proposed method could reduce the errors in path following in ARP. The proposed method can be applied in real scenarios and to develop a model to optimize ARP systems for AVs. Similarly, the proposed method can provide a reference for future extensive research such as forward parking and parallel parking, and can be implemented in the navigation of mobile robots in crowded and cluttered environments.

## Figures and Tables

**Table 1:** Related studies on autonomous rear parking.

Related Studies	Objectives and Description	Methods (○: Partially Used, ●: Fully Used)			
		RRT	MPC	MDP-Based RL	OpenAI Gym
[8,10]	Local path planning	●			
[11,12]	Path following and motion controller	●	○		
[9,13]	Mathematical model for decision making	●	○	●	
[6,14]	Training the agent to adapt to the environment based on human or expert demonstration	●	●	○	
[7,15]	Sensitivity-based path-following approach	●	●	○	
[16,17]	Actor–critic-based Q-learning with deep neural network	●	●	●	
Proposed method	ARP based on path planning, predictive control, robust decision making, and training of the agent	●	●	●	●

**Table 2:** Classification of linear and non-linear MPC.

Linear MPC	Non-linear MPC
1. Uses a linear model: $x=A_{x}+B_{u}$ 2. Quadratic cost function: $F=x^{T}+Q_{x}+u^{T}Ru$ 3. Linear constraints: $H_{x}+G_{u}<0$ 4. Quadratic program.	1. Non-linear model: x=f(x, u) 2. Cost function can be nonquadratic: F=(x, u) 3. Non-linear constraints: h(x, u)<0 4. Non-linear program

**Table 3:** Parameters for experimental vehicle and environment.

	Component	Parameter
Vehicle parameters	Width	18.2 cm
	Wheelbase	19.8 cm
	Length	28.9 cm
	Distance between front and rear wheel axles	2.6 cm
	Minimum turning radius	34.02 cm
Environment parameters	Number of parking slots	4
	Number of available parking slots	1
	Environment boundary	(*x*, *y*) = (100, 100)

**Table 4:** Quantitative analysis used to calculate the accuracy using only MPC.

Scenario	Total Error Count	Total Error Distance Estimation $(\mathrm{Error}=\surd {\left(x_{2}-a_{2}\right)}^{2}$ + ${\left(y_{2}-b_{2}\right)}^{2})$	Error % Error % = Total Error Distance Estimation/Total Error Count	Accuracy (%)
Case 1	8	1.24	15.5%	84.5%
Case 2	14	1.19	8.5%	91.5%
Case 3	13	1.09	8.38%	91.62%
Case 4	14	1.02	7.28%	92.72%
Case 4	15	1.01	6.7%	93.3%
Case-6	11	1.12	10.18%	89.82%
		Average	9.42%	90.58%

**Table 5:** Quantitative analysis used to calculate the accuracy using the proposed method (RL).

Scenario	Total Error Count	Total Error Distance Estimation $(\mathrm{Error}=\surd {\left(x_{2}-a_{2}\right)}^{2}$ + ${\left(y_{2}-b_{2}\right)}^{2})$	Error % Error % = Total Error Distance Estimation/Total Error Count	Accuracy (%)
Case 1	6	0.26	4.33%	95.67%
Case 2	11	0.7	6.33%	93.67%
Case 3	10	0.64	6.4%	93.6%
Case 4	9	0.38	4.22%	95.78%
Case 4	11	0.79	7.18%	92.82%
Case 6	9	0.41	4.55%	95.45%
		Average	5.50%	94.50%

**Table 6:** Comparison of experimental results between proposed ARP system and existing approaches.

References	Methods	Computational Time (s)	Error (%)	Accuracy (%)
Zhang et al. [23]	A*, optimization-based collision avoidance	13.86 s	15.80%	84.20%
Zhang et al. [24]	Breadth-first, Bellman–Ford algorithm	12.72 s	13.30%	86.70%
Table 3	MPC	10.99 s	9.42%	90.58%
Table 4	Proposed method	4.33 s	5.50%	94.50%
